# Vehicular Manslaughter: The Global Epidemic of Traffic Deaths

**DOI:** 10.1289/ehp.112-a628

**Published:** 2004-08

**Authors:** Richard Dahl

Even though traffic-related death rates in the United States and other high-income countries have been declining steadily for several decades, death tolls on the roadways of the world’s poorer countries have been skyrocketing. The number of motorized vehicles has been escalating in developing countries, where roads (often poorly built to begin with) are shared by pedestrians, animal-driven carts, rickshaws, and bicycles, and traffic safety laws are weak or inadequately enforced. The result—an alarming increase in death and injury on the roadways of the world’s poorer nations—is now attracting the attention of international organizations as a major public health problem.

In recognition of this global problem, the World Health Organization (WHO) designated “road safety” as the theme of World Health Day 2004, held April 7. The same day, the WHO and the World Bank released the *World Report on Road Traffic Injury Prevention*, an exhaustive examination of the global problem and potential solutions, culminating in a call for action at both the national and international levels. A week later, the United Nations (UN) General Assembly devoted a plenary session to the global crisis in road safety for the first time in its history, discussing, among other things, how to implement the report. Then, in May 2004, at the WHO 57th World Health Assembly meeting in Geneva, delegates accepted resolution EB113.R3, “Road Safety and Health,” calling for the WHO to act as a coordinator on road safety issues within the UN system, working in close collaboration with UN regional commissions.

As the WHO/World Bank report makes clear, the increase in traffic deaths and injuries in the world’s poorer nations has been evident for years: global traffic deaths have risen from approximately 990,000 per year in 1990 to nearly 1.2 million per year in 2002, with 85–90% of these deaths occurring in low- and middle-income countries. Citing a study that examined changes in traffic fatality rates in various countries between 1975 and 1998, the report noted that during that time span Canada’s road fatality rates declined by 63.4%, Sweden’s by 58.3%, and those in the United States by 27.2%; the reasons for these declines have been multifold. At the same time, traffic fatalities increased by 237.1% in Colombia, by 243.0% in China, and by 383.8% in Botswana.

Furthermore, the report predicts that if nothing is done to stop the trend, it will rapidly escalate. By 2020, predict the WHO and the World Bank, if appropriate actions are not taken, overall global traffic deaths will increase by 67%; an 83% increase in poorer countries will offset a projected 30% decrease in high-income countries. In 1990, road traffic injuries were the ninth leading contributor to the global burden of disease, according to the report. Without appropriate action, by 2020 road injuries are predicted to be the third leading contributor.

Many physicians and public health professionals have been sounding the alarm for years and voicing frustration over the lack of international response. Samuel N. Forjuoh, a Ghanaian-born physician and professor of family and community health at Texas A&M University College of Medicine, believes that part of the problem has been that road crashes are simply not seen in the same way as diseases. “For a long time, injuries were considered haphazard events, acts of God,” he says. “There was a belief that there was nothing you could do about them.”

Another reason is that car crashes occur one at a time, says Mark L. Rosenberg, executive director of the U.S.-based nonprofit Task Force for Child Survival and Development and former director of the Centers for Disease Control and Prevention’s (CDC) National Center for Injury Prevention and Control. “You have three thousand people a day dying in car crashes,” he explains. “If you had three thousand people a day dying in one building that collapses or in a few big airplane crashes, it would get much bigger headlines.”

Still another reason is a sense of fatalism on the part of some observers. Rosenberg explains, “People say, ‘Oh, motor vehicle deaths are just what happens when you start development in a country. You build roads, you bring in cars, and this is inevitable.’ But nothing could be further from the truth. It’s not inevitable; [these deaths] are completely predictable and preventable.”

## A Perfect Plague

Rosenberg borrows from the popular book and movie *The Perfect Storm*, which told how a rare combination of meteorological forces resulted in a monstrously destructive force, to describe the escalation in traffic deaths and injuries in poorer countries. “More and more motorized vehicles are being added to roadways that are inadequate,” he says. “You have these mixes of vulnerable pedestrians and motorized traffic on main roads with no center barriers. The roads are poorly marked, and they go right through villages. You have situations where people don’t know how to drive, they don’t obey laws, and even if you have laws, they’re not enforced. So you have this lethal mix, and into this we are adding more and more cars, accelerating the rate of injury and death as these cars are added. You have the ‘perfect plague.’” Rosenberg says this plague is also perfectly predictable because “we know how quickly the motor vehicle manufacturers are planning to increase their production in these ripest of markets.”

According to the *World Report*, a large portion of the motor vehicle increase in poorer countries has been in the number of motorcycles, minibuses, and trucks. And their increased presence on the roadways of those countries has been overwhelming in many instances. Since 1990, the number of motor vehicles in China, for instance, has quadrupled to more than 55 million. In Thailand, the number of motor vehicles nearly quadrupled between 1987 and 1997.

The new and growing mix of two-wheeled motor vehicles, larger multi-passenger vehicles, traditional wheeled vehicles, and pedestrians all often using the same roadways has created patterns of traffic death and injury that are much different from those in high-income countries. The WHO/World Bank report points out that while most of the people who die as the result of traffic crashes in the developed world are passengers in vehicles, traffic crashes in poorer countries are more likely to involve pedestrians and motorcyclists. The report states that between 1977 and 1994 in the city of Nairobi, for instance, 64% of traffic fatalities were pedestrians.

Another characteristic of poorer countries is that crashes are far more likely to result in deaths than crashes in high-income countries. Rosenberg explains, “The victims [in developing nations] are much, much more vulnerable: they are pedestrians or bike riders with nothing to protect them.” Further, says Michael R. Reich, a professor of international health policy at the Harvard School of Public Health, the typical U.S. car crash involves the driver running into a tree or into another vehicle. In contrast, crashes in poor countries typically involve pedestrians or people in poorly maintained multi-passenger vehicles, such as mini-buses crammed with 20–30 people and no seat-belts. According to “Road Traffic Injuries in Developing Countries: Strategies for Prevention and Control,” a resource paper by Reich and Harvard colleague Vinand Nantulya, 10,000 U.S. crashes result in 66 deaths; but in Kenya, the death rate per 10,000 crashes has been as high as 1,786, and in Vietnam it’s reached 3,181.

A variety of other factors lend to the troubling numbers in poor countries. Forjuoh believes that low literacy rates play a role because many drivers can’t understand road signs. David Sleet, associate director for science at the CDC National Center for Injury Prevention and Control and a coeditor of the WHO/World Bank report, contends that many pedestrians in developing countries have never driven a car and don’t understand the basic “rules of the road” governing motorists. They also are inexperienced in judging oncoming vehicle speeds and stopping distances.

Yet another factor exacerbating the traffic fatality trend in poor countries is the shortage—and often the absence altogether—of emergency medical response. Charles N. Mock, a surgeon and epidemiologist at the University of Washington’s Harborview Injury Prevention and Research Center in Seattle, has spent time in Ghana and has worked with groups in Mexico, Vietnam, and India to improve systems of emergency medical care in those nations. He points out that while organizations such as Doctors Without Borders and the International Committee of the Red Cross can provide physicians on episodic bases, such as in response to earthquakes and wars, there really isn’t an available pool of doctors that poor countries can call upon to provide sustained improvement of emergency medical response.

Mock admits that the situation may sound dismal, at least at the moment. “But to sit back and say nothing can be done because of the economic problems isn’t fruitful,” he says. “A tremendous amount can be accomplished. Mortality rates can be decreased, medically preventable death can be prevented, disabilities can be prevented, and part of the discrepancies in outcome between rich and poor countries can be eliminated by improving organization and planning for trauma care services without necessarily spending very much more.”

## Sensible Responses to the Epidemic

Mock has been intimately involved in a project, cosponsored by the WHO and the International Society of Surgery, to formulate a low-cost model that nearly every country can implement to greatly improve emergency trauma care. The model identifies specific essential elements that are necessary for trauma care—human resources, equipment, supplies—for purposes of aiding countries in performing needs assessments. Mock has spent this year at WHO headquarters in Geneva working on the plan’s implementation.

Other organizations are embarking on other programs. In 1999, the World Bank created an organization called the Global Road Safety Partnership (GRSP), an international collaboration between business, civil society, and government organizations to improve road safety conditions around the world. GRSP is currently involved in a variety of projects in 10 countries. For example, in Bangalore, India, GRSP has created partnerships to launch an anti–drunk driving campaign and to improve roadways in high-traffic areas to enhance safety. In addition, the CDC has been working with the ministries of health and other groups in Mexico, Colombia, and El Salvador to devise creative new ways to reduce injuries to pedestrians, bicyclists, and motor vehicle occupants. And the World Bank is providing $25 million for the Vietnam Road Safety Project with a goal of achieving continuous, long-term reduction of traffic crashes in that country.

Sleet also points to other local strategies in place that reduce the likelihood of crashes. Some cities are pursuing better land use management for optimized traffic flow, and promoting alternative transportation modes such as mass transit. Other strategies target drunk driving and speeding behaviors, and promote the use of cycle helmets, seat belts, and other protective devices. Still others seek to separate non-motorized traffic (such as pedestrians and cyclists) from motorized traffic.

While these efforts are noteworthy, reducing the epidemic of traffic deaths and injuries around the globe will be impossible unless countries adopt the political will to do so on their own, states the *World Report*. In the United States, for instance, the will to do something about the nation’s ever-growing highway death tolls began in the 1960s. Ralph Nader’s 1965 book *Unsafe at Any Speed* detailed the minimal attention being paid to safety in the auto industry, and the next year President Lyndon Johnson signed two bills to create stricter safety standards in cars and roadways. The combination of safer cars and roadways coupled with successful efforts to promote safer behaviors (such as use of seat belts and “designated drivers”) has been credited with bringing about ever-diminishing rates of traffic death and injury on U.S. roadways.

While the United States and other developed nations possess the resources to invest in safer cars, better roadways, and improved emergency medical response, an obvious question is, how can poorer countries achieve improvements as well? According to Sleet and others who are knowledgeable about traffic safety, the answer is by adopting and adapting effective strategies, and by developing local evidence-based solutions.

In Colombia, for instance, a concerted effort to reduce traffic deaths in Bogotá and other cities has proven to be extremely successful. In 1995, Colombia first required that all vehicle owners must be insured and then instituted a 3% levy on all vehicle insurance policies, earmarking that money for a “road accident prevention fund.” Colombia had recorded its all-time record high for traffic fatalities—7,874—in 1995, according to a report in the March–June 2003 issue of *Injury Control and Safety Promotion* by a team led by Deysi Yasmin Rodríguez, an engineer with the Research Program on Traffic and Transport at the National University of Colombia. By 2002, the nation’s traffic deaths had dropped to 6,063.

In Bogotá, the nation’s capital and biggest city with 7 million inhabitants, a series of mayors have instituted several programs to reduce traffic deaths and injury, such as closing bars at 1 a.m. instead of 3 a.m. and urging people to drink in moderation if they’re driving, reducing the number of cars during rush hour by encouraging workers to find alternative means of transportation (such as carpooling), and reclaiming sidewalks for pedestrians by prohibiting the old practice of allowing drivers to park their cars there. The outcome has been striking: the number of traffic fatalities in Bogotá declined from 1,387 in 1995 to 697 in 2002.

According to Rodríguez, the national Ministry of Transport is now finishing plans to broaden the Bogotá approach to the entire nation. “Among the main strategies we have is the generation of a new culture about road safety in the country,” says Rodríguez, who is a consultant on the project.

Still, even though Colombia has made great progress, Rodríguez sees distinct obstacles ahead. There are “few economic and human resources capable of supporting the programs in a continuous way,” she says.

The WHO/World Bank report includes a series of recommendations to reduce global traffic deaths and injuries. It strongly advocates the identification of a lead agency in every nation to be in charge of traffic safety. It then recommends the routine collection of traffic crash and injury data to document the magnitude of the problem and the key risk factors. It goes on to recommend the formulation of a national plan of action in every country, together with implementation of the plan using science-based interventions.

But as Rosenberg emphasizes, there are serious impediments to the creation of good traffic safety plans in poor countries. “We say, ‘Form a coordinating agency in your country,’ but we’ve got to get them the resources to do it,” he says. “Each step takes money, and right now they don’t have the resources.”

To Rosenberg, the problem is akin to where AIDS/HIV stood 20 years ago. “We tended to the problem just within our own borders and completely ignored what was happening in sub-Saharan Africa until it was too late. We need to learn from that epidemic and apply it to this one, because with this one we have a chance to prevent it,” he says. “When AIDS came twenty years ago there were no good means to prevent it or treat it, and not even a good test to diagnose it. But with traffic safety there are many very clear and effective things we can do. If we let this one get out of control, we will have no excuse. We will have failed in our responsibility to become good ancestors.”

## Figures and Tables

**Figure:**
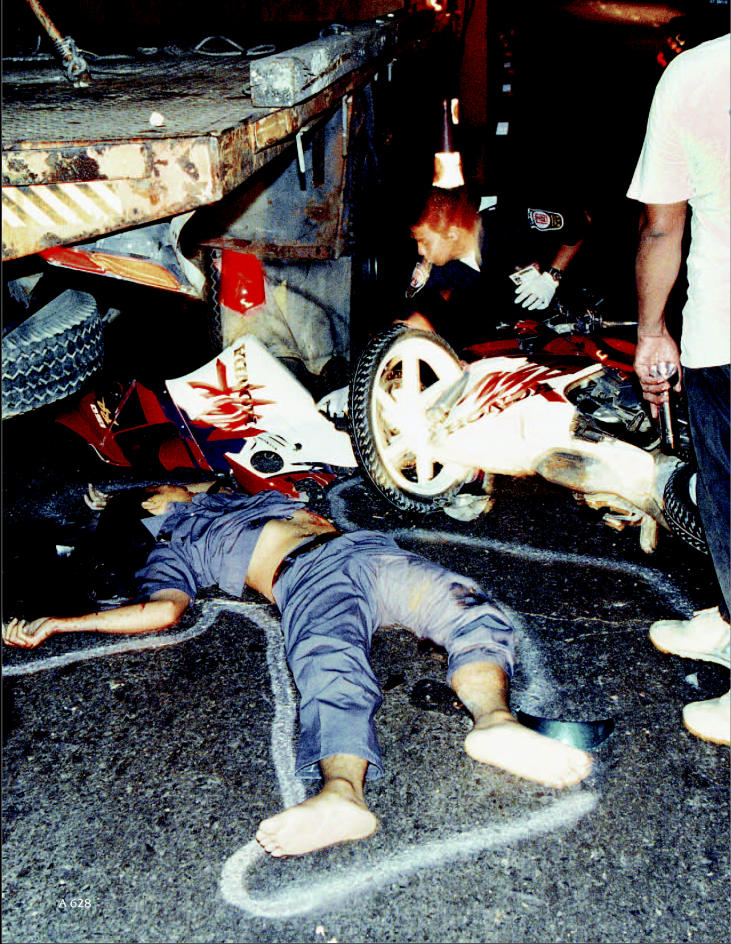
**Collision course.** Traffic crashes such as this one in Bangkok, Thailand, resulted in 1.2 million deaths worldwide in 2002, with as much as 90% of those occurring in low- and middle-income countries.

**Figure:**
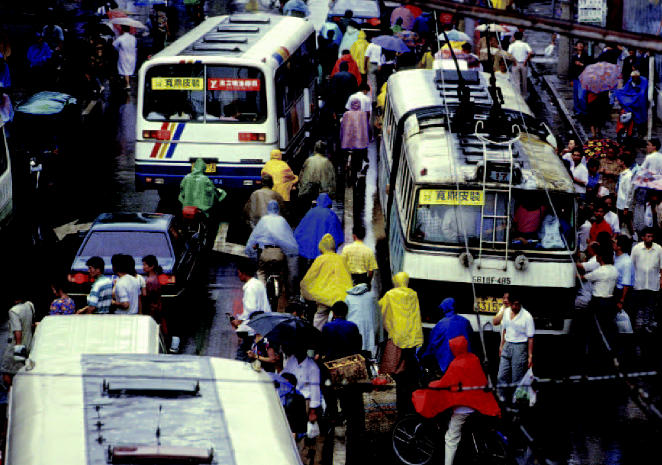
**Street fight.** Scenes like this one of congested vehicle and pedestrian traffic in Shanghai are an everyday occurrence worldwide as humans and machines compete for use of the roads.

